# Development of a Sensitive Method for Cadmium Determination in Fish Tissue and Drinking Water Samples by FAAS Using SQT In Situ Atom Trapping

**DOI:** 10.1021/acsomega.2c07926

**Published:** 2023-02-13

**Authors:** Hüseyin Karababa, Muhammet Atasoy, Dilek Yildiz, İbrahim Kula, Mustafa Tuzen

**Affiliations:** † Department of Chemistry, Muğla Sıtkı Koçman University, Menteşe 48000 Muğla, Turkey; ‡ Muğla Vocational School, Chemistry and Chemical Treatment Technologies Department, Chemistry Technology Program, Muğla Sıtkı Koçman University, Menteşe 48000 Muğla, Turkey; § Environmental Problems Research and Application Center, Muğla Sıtkı Koçman University, Menteşe 48000 Muğla, Turkey; ∥ Faculty of Arts and Sciences, Department of Chemistry, Tokat Gaziosmanpaşa University, 60250 Tokat, Turkey

## Abstract

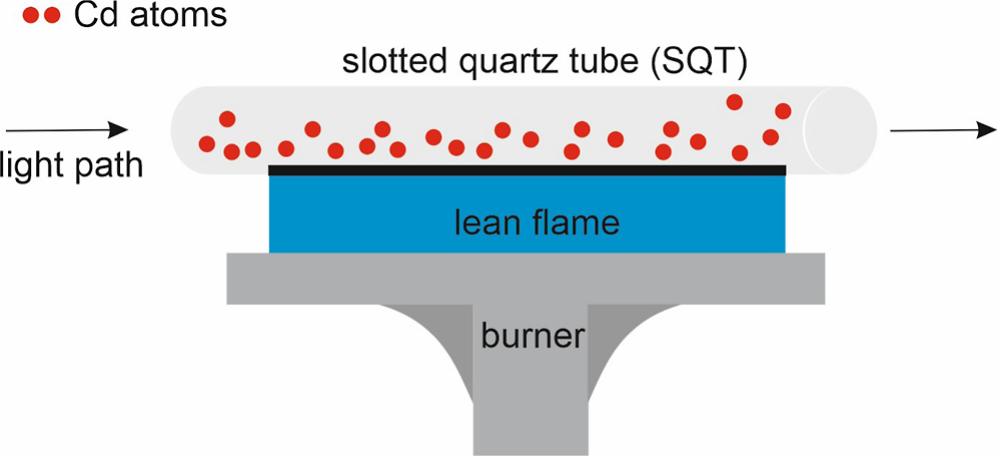

A sensitive and robust trap method was developed for the determination of cadmium (Cd) by using a slotted quartz tube. Using this method at a sample suction rate of 7.4 mL/min for 4.0 min collection, a 1467-fold increase in sensitivity was obtained compared to the flame atomic absorption spectrometry method. Under the optimized conditions, a limit of detection of 0.075 ng mL^–1^ was obtained for the trap method. The interference effects of hydride-forming elements, transition metals, and some anions on the Cd signal were investigated. The developed method was evaluated by analyzing “Sewage Sludge-industrial origin (BCR no: 146R)”, “NIST SRM 1640a Trace elements in natural water”, and “DOLT: 5 Dogfish Liver”. There was a good agreement between the certified and found values at the 95% confidence level. This method was applied successfully for the determination of Cd in drinking water and some fish tissue samples (liver, muscle, and gill) obtained from Muğla province.

## 1 Introduction

One of the most important problems in today’s world is the pollution of drinking water and water ecosystem, especially due to industrial activities.^1,2^ Cadmium (Cd) has important uses in the industrial field. It is used in the electric industry and galvanization, polyvinyl chloride production, and plastic and glass industry and as a pigment. In addition, it is used as a cathode material in the Ni–Cd battery industry.^3^ It has been identified as one of the most dangerous trace metals in the environment due to its widespread distribution.^4^ The main source of Cd pollution in the environment is anthropogenic activities.^5^ Cd is one of the elements with the highest risk of damaging human organs such as the kidney, testis, lung, liver, and brain, even at low concentrations.^1^ The fact that it has a biological half-life of 10–30 years shows that it is permanent in the human body. Therefore, it is one of the most toxic elements for the human body.^6^ It is considered as an extremely important pollutant as it is highly soluble in water.^7^ It is considered as a potential carcinogen.^8^ Regular exposure to Cd causes accumulation in the body and increases the risk of osteoporosis and cancer.^9^ When the concentration of Cd accumulated in the kidney exceeds 200 mg kg^–1^, its functions are damaged.^10^ Cd is an element with high mobility in the soil, and it can be easily accumulated by plants.^11^ Thus, it can get into the animal food chain or contaminate the aquatic environment by being washed from the soil. This situation disrupts the balance of environmental conditions and affects food security and poses a threat to human health.^12^ Additionally, Cd is carried to the lower layers of the soil by chelating agents, and it contaminates the underground water and causes heavy metal pollution in drinking and irrigation waters.^13^ Environmental Protection Agency (EPA) allowed the maximum contaminant level of Cd in standard drinking water to be 5.0 μg L^–1^.^14^ In addition, determination of trace levels of Cd in food samples has received great interest in public health and environmental issues.^15^ Fish plays an important role in the human diet as it is an excellent source of proteins, lipids, fatty acids, minerals, and vitamins.^16^ Fish samples are suitable bioindicators for the estimation of the potential for heavy metal pollution in aquatic systems because fish are located at the top of the food chain in aquatic systems.^17,18^ Fish are at the upper levels of the food chain and can accumulate heavy metals in water at high concentrations.^19^ 0.05 mg kg^–1^ has been determined by the European Union as the maximum Cd level in fresh fish.^20^ The improvement of simple, accurate, and robust analytical methods for the monitoring and detection of trace levels of Cd in environmental, biological, and food samples is very significant to prevent human and animal exposure to this toxic element.^8^ Prior to the determination of the trace or ultratrace level analyte concentration in the complex matrix, there is a need for preconcentration techniques that can achieve low detection limits and eliminate or minimize matrix effects.^14^

Inductively coupled plasma mass spectrometry (ICPMS),^21^ inductively coupled plasma emission spectrometry (ICPOES),^22^ and atomic fluorescence spectrometry (AFS)^23^ have been performed for the determination of Cd in environmental and biological samples.^24^ These methods are widely used because precise results and low detection limits can be obtained.^25^ However, the instruments used in these methods are very expensive, and the operating costs of these instruments are very high.^26^ Another technique is atomic absorption spectrometry (AAS),^27,28^ and AAS is a robust spectrometric method that has been used for many years. Determination of ultratrace Cd concentration is very important in environmental and biological samples. It is not possible to directly determine at the trace or ultratrace level of Cd concentration with flame AAS (FAAS). To increase the sensitivity of FAAS, atom traps have been developed.^29^ The atom trap may be a quartz surface or a W-coil. The analyte species are trapped on a quartz or a W-coil in the collection period before the re-volatilization processes to obtain a sensitive analytical signal.^30^ For enhancement of residence time of analyte atoms in the optical path, the long-path absorption tube^29^ and slotted quartz tube (SQT)^31^ are used. In addition, the U-tube atomic trap,^31^ the integrated atomic trap,^29^ and the SQT atomic trap (SQT-AT)^32^ are the techniques used for preconcentration. In the SQT-AT technique, the analyte atoms are preconcentrated on the inner surface of the SQT.^32^ After a sample is sent to the system for a certain period of time, organic solvents are introduced to the system for revolatilization of the collected analyte species on the quartz surface. Organic solvents increased the flame temperature immediately for a short time.^30,33^ The most important advantage of the SQT-AT technique is that it provides a significant increase in sensitivity. In addition, since the analyte atoms are preconcentrated, the matrix effect is reduced.^34^ Its low cost and simplicity of use are other important advantages. The principal disadvantage of the technique is that it is limited to only volatile elements.^35^

The purpose of this work is to develop a simple, sensitive, and rapid technique for the determination of Cd in water and fish tissue samples. To the best of our knowledge, Cd concentration in fish tissue samples was determined for the first time by the SQT-AT technique. This technique is based on the trapping of Cd atoms on the inner surface of the SQT. The proposed trap technique was validated by the analysis of certified reference materials. Interferences of some transition metals, hydride-forming elements, and anions were also investigated.

## 2 Experimental Section

### 2.1 Chemicals and Reagents

All reagents used in the study were at least of analytical reagent grade. Standard solutions were prepared by appropriate dilution of the 1000 mg L^–1^ Cd stock solution. Dilutions were made using ultrapure water obtained from a Milli-Q Plus water purification system (Millipore, Bedford, USA, 18.2 MΩ cm). For the acidification of solutions, 65% (w/w) HNO_3_ (Merck) was used. All standard solutions used in experimental studies were prepared in 1.0 mol L^–1^ HNO_3_. All glass and polyethylene containers were kept in a cleaning solution containing 10% HNO_3_ for at least 1 day and then washed with deionized water. To check the accuracy of system for Cd determination, Sewage Sludge-industrial origin (BCR no:146R), DOLT: 5 Dogfish Liver, and NIST SRM 1640a “Trace elements in natural water” standard reference materials were used.

The effect of every interferent elements was investigated by preparing standards with Cd/interference (w/w) ratios of 1:1, 1:10, and 1:100. During the interference study, the Cd concentration was kept constant at 20.0 ng mL-1. Ni (Merck), Fe (Merck), Zn (Merck), Mn (Merck), Se (Merck), and As (Merck), were prepared from their 1000 mg L^–1^ stock solutions. Furthermore, interference effects of some anions such as PO_4_^3–^ prepared from KH_2_PO_4_ (Riedel-de Haen), NO_3_^–^ prepared from Ca(NO_3_)_2_ (Merck), Cl^–^ prepared from KCl (Merck), and SO_4_^2–^ prepared from Na_2_SO_4_ (Merck) were investigated on the Cd signal.

**Figure 1:**
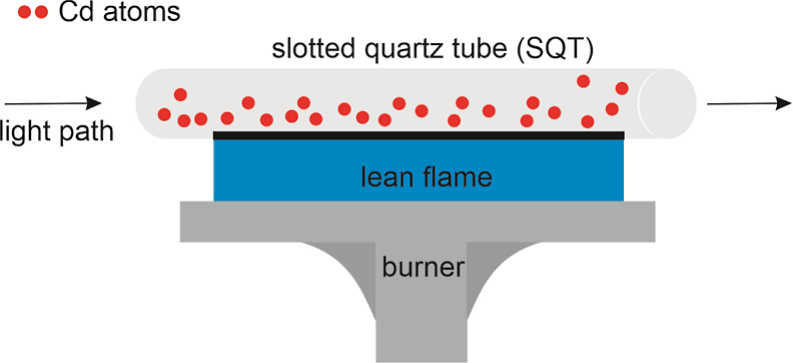
Schematic diagram of the SQT on the burner head of the instrument.

An SQT with a length of 15 cm, an inner diameter of 13 mm, and an outer diameter of 17 mm, with an angle of 180° between the slits, was used. The SQT was made by OSTİM (Middle East Industry and Trade Center) Çalışkan Cam. This SQT was placed on the burner head of the instrument by the handmade apparatus aligned to the optical beam of the instrument. The schematic diagram of the SQT on the burner head of instrument is given in Figure 1.

### 2.2 Instrumentation

All measurements were carried out with GBC Avanta Sigma 906 Model AAS equipped with a deuterium background correction system. The wavelength adopted for the measurement of Cd was 228.8 nm. The Cd hollow cathode lamp was operated at 4.0 mA, and spectral band pass was 0.2 nm. The length of the burner head was 100 mm. Air/acetylene flame was used. The air flow rate and acetylene flow rate were 12.0 and 1.5 L min^–1^, respectively. The measurements were based on the peak height absorbance.

### 2.3 Procedure for SQT-AT-FAAS

The SQT was placed on the burner; then, a lean flame was obtained with a 1.1–12.0 L min^–1^ air–acetylene. Thus, a suitable flame medium was created in order to absorb the analyte atoms on the inner surface of the quartz tube. The Cd sample was nebulized on the flame. Cd atoms were trapped to the inner surface of the SQT for 3 min. After the collection step, 40 μL of the volume organic solvent [methyl isobutyl ketone (MIBK)] was sent to the system, and analyte atoms were revolatilized. Finally, the transient analytical signal was obtained, and its peak height was used for signal measurement.

### 2.4 Sample Preparation

Drinking water samples were provided from Muğla province and acidified to contain 1.0 mol L^–1^ HNO_3_. Fish samples were collected by Akyaka Aquaculture Cooperative, and these were among the highly selected species for consumption by the local population. Muscle, gill, and liver tissues of all fish samples were identified. Approximately 0.1–0.5 g of each fish tissue sample was placed in Teflon vessels containing 10 mL of HNO_3_ (65%). Samples were digested with the CEM Mars 6 microwave digestion system as proposed by Atasoy et al.^36^ DOLT:5 and BCR 146R standard reference materials were also digested using the same digestion process.

## 3 Results and Discussion

First, Cd was determined using the FAAS method. Second, Cd determination was carried out by the SQT-FAAS method. The SQT was used to increase the residence time of analyte atoms in the optical path. Thus, two to five times increased sensitivity was achieved. No trapping was performed in this method. Third, the SQT-AT-FAAS method was used to improve the sensitivity of FAAS for Cd determination by creating a simple, inexpensive, and easy way to the SQT for atom trap. This method involves trapping analyte atoms on the inner surface of the SQT. After the trapping step, Cd atoms were released from the surface of the SQT by aspiration of an organic solvent. Primarily, parameters that could affect the Cd signal were optimized for all the three methods. A univariate optimization was carried out.

### 3.1 Determination of Cd by the FAAS Method

In this method, acetylene flow rate optimization was performed before obtaining the calibration graph of Cd in flame AAS and determining the LOD and LOQ values. The study was performed with 1.00 mg L^–1^ Cd solution. The air flow rate was kept constant at 13.8 L min^–1^, and the most appropriate absorbance was obtained by changing the acetylene flow rates. Optimum acetylene flow rate was determined as 2.3 L min^–1^.

To create the calibration graph, a series of Cd solutions were prepared between 0.1 and 100 mg L^–1^ concentrations, and the absorbance values were measured. Linearity was observed between 0.25 and 5.0 mg L^–1^. LOD and LOQ values were calculated with the standard deviation obtained by reading the blank solution 13 times. LOD and LOQ values were obtained as 110 and 366 ng mL^–1^, respectively.

### 3.2 Determination of Cd by the SQT-FAAS Method

As in FAAS, the acetylene flow rate was optimized in the determination of Cd with an SQT. The air flow rate was again kept constant at 13.8 L/min, and the optimum absorbance was obtained by varying the acetylene flow rates. Optimization was carried out with 1.00 mg L^–1^ Cd solution. The optimum value of acetylene flow rate was obtained as 1.5 L min^–1^.

To create the calibration graph, Cd solutions were prepared at increasing concentrations between 5.0 ng mL^–1^ and 5.0 mg L^–1^, and absorbance values were obtained. The linearity range was determined between 0.025 and 1.0 mg L^–1^. LOD and LOQ values were calculated as 5.0 and 16.5 ng mL^–1^, respectively.

### 3.3 Determination of Cd by the SQT-AT-FAAS Method

In this method, Cd atoms are trapped on the inner surface of the SQT. The trap method consists of three steps: collection, revolatilization, and atomization. In the collection step, the sample solution is nebulized onto the flame in an optimized lean flame media. For a few minutes, Cd atoms are trapped on the inner surface of the SQT. In the revolatilization step, an organic solvent such as MIBK or methyl ethyl ketone with a low volume of 10–50 μL is sent into the flame. As soon as the organic solvent reaches the flame, the flame changes its composition for a very short time and Cd atoms are released from the inner surface of the SQT. In the last step, atomization takes place, and a transient signal is obtained at this step.

Parameters that could affect the analytical signal of Cd such as flame conditions and suction rate of the sample were optimized. In addition, a suitable organic solvent was selected to obtain maximum trapping efficiency with the SQT-AT-FAAS method. The volume of the selected organic solvent and trapping time were also optimized. All optimizations were performed using 2.0 ng mL^–1^ Cd standard solution.

A highly flammable organic solvent is needed for rapid revolatilization of analyte atoms. Various organic solvents such as MIBK, acetonitrile, acetone, and *n*-hexane were used. Other conditions were kept constant, and only organic solvents were changed. Cd standard solution was trapped on the inner surface of the quartz tube for 3 min, and the analytical signal of Cd was obtained by using the same volume of organic solvents. All the organic solvents used also allowed the revolatilization of the analyte atoms, but the highest signal was obtained with MIBK. Therefore, MIBK was chosen as the optimum organic solvent. It was concluded that a small volume of the organic solvent (10–50 μL) was sufficient for revolatilization of all the analyte atoms. The optimum organic solvent volume was determined as 40 μL. No significant increase in the Cd signal was obtained when higher volumes of the organic solvent were used. Moreover, the flame went out from the ends of the SQT as the intensity of the flame suddenly increased when a high volume of the organic solvent was used. This situation caused pollution on both window surfaces where the beam on the right and left of the SQT passed.

The flow rate of acetylene was optimized in the SQT-AT method in this study. The air flow rate was kept constant at 12.0 L min^–1^. The intense flame media facilitated atomization but prevented the trapping of analyte atoms on the surface of the SQT. In order to absorb the Cd atoms onto the inner surface of the quartz tube, the intensity of the flame was reduced in the trap method.^33^ Therefore, a weak, low-fuel flame was used.^25^ The optimum acetylene flow rate was found to be 1.1 L min^–1^.

It was observed that when the analyte atoms were trapped for a longer time, the analytical signal of Cd increased, thus increasing the sensitivity. Trapping for a longer time increased sample and chemical consumption. The optimum collection time was chosen as 4 min, since no more trapping time was needed.

### 3.4 Interference Studies on the Cd Signal with the SQT-AT-FAAS Method

**Figure 2:**
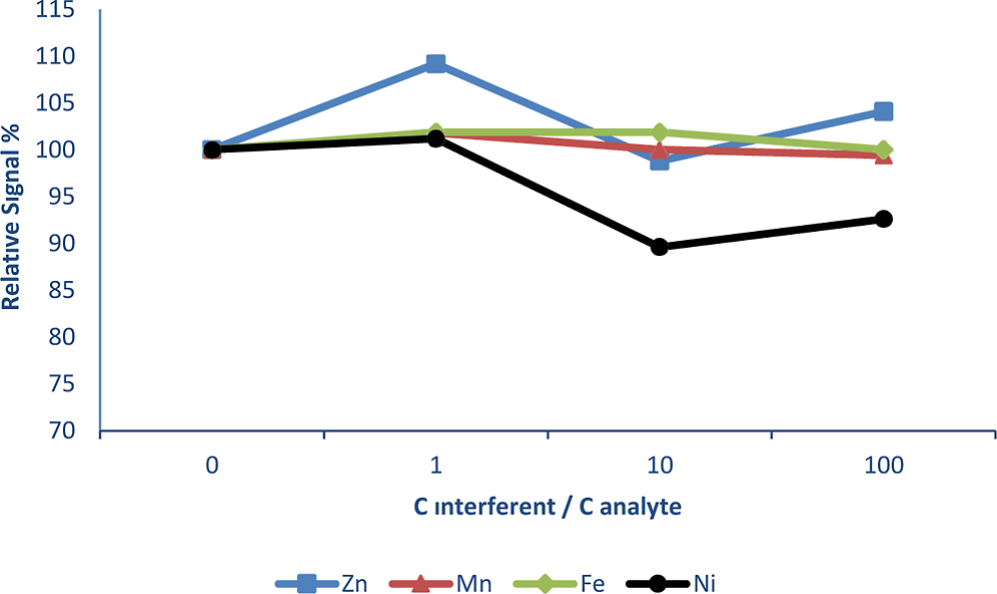
Interference effect of transition metals on Cd.

**Figure 3:**
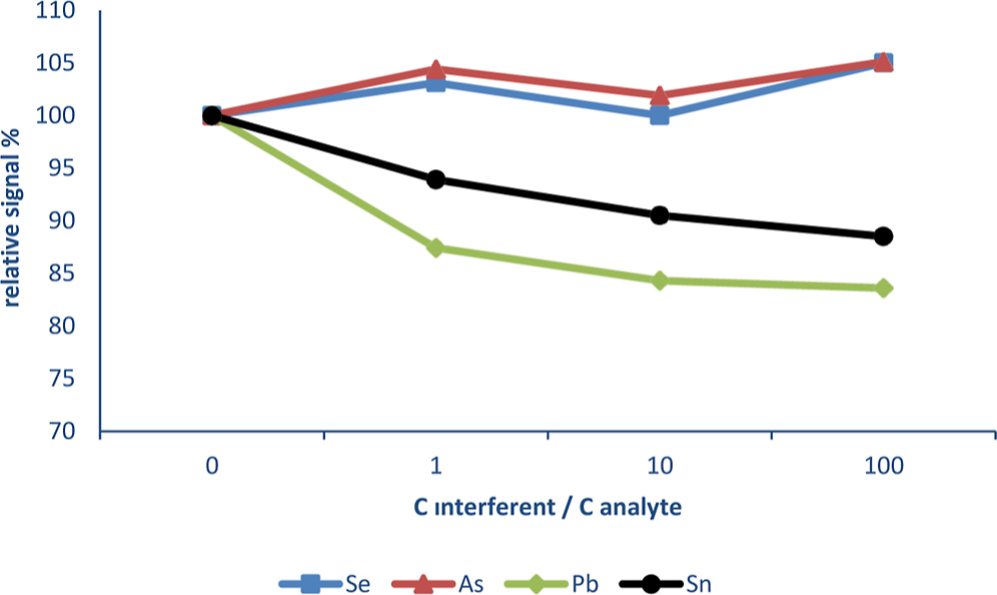
Interference effect of hydride-forming elements on Cd.

**Figure 4:**
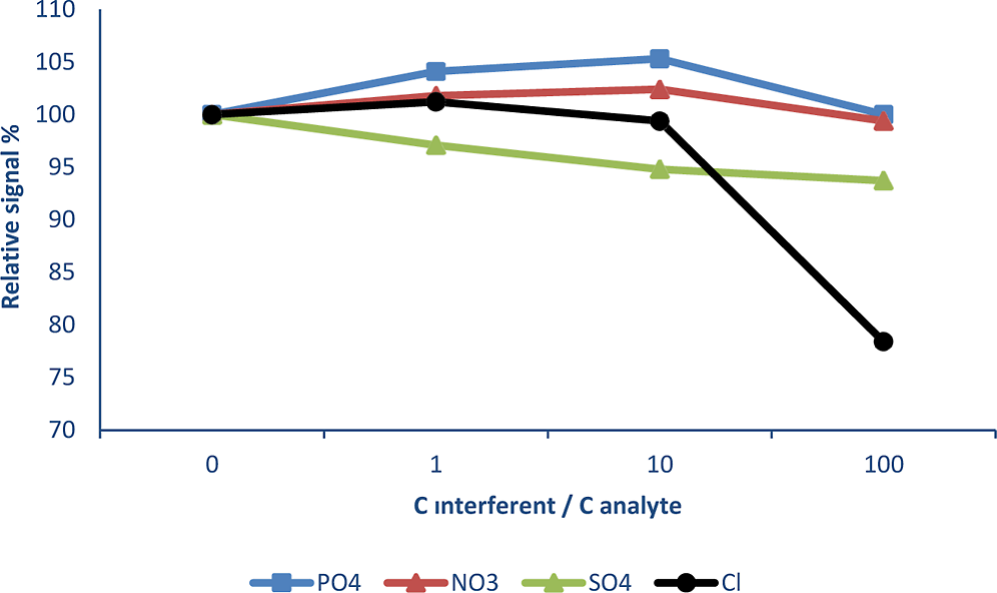
Interference effects of some anions on Cd.

During this study, interference effects of some transition metals (Ni, Fe, Zn, and Mn), hydride-forming elements (Se, As, Pb, and Sn), and anions (PO_4_^2–^, NO_3_^–^, Cl^–^, and SO_4_^2–^) on the Cd signal were investigated using the SQT-AT-FAAS method at a collection period of 4.0 min, and results are shown in Figures 2–4 below, respectively. For this aim, three different solutions were prepared in which the concentration of Cd was kept constant as 0.1 mg L^–1^, and concentrations of interferents were 1-, 10-, and 100-fold analyte concentration, using the mass ratios. In Figure 2, interference effects of the transition elements on the Cd signal are given. Zn caused a sensitivity increase by about 9.2% when the interference/analyte ratio was 1. In addition, Ni caused a sensitivity decrease by about 10.4 and 7.4% when the interference/analyte ratio was 10 and 100, respectively. Other transition elements did not cause significant interference.

In Figure 3, the interference effects of hydride-forming elements are shown. Pb reduced the signal of Cd at all the studied concentrations. Pb decreased the Cd signal by 12.6, 15.7, and 16.4% when the interference/analyte ratio was 1, 10, and 100, respectively. In addition, Sn also caused a sensitivity decrease by about 9.5 and 11.5% when the interference/analyte ratio was 10 and 100, respectively. Other hydride-forming elements did not cause significant interference.

In Figure 4, interference effects of some anions such as PO_4_^3–^, NO_3_^–^, SO_4_^2–^, and Cl^–^ are given. Cl^–^ caused a sensitivity decrease by about 21.6% when the interference/analyte ratio was 100. Other anions used in this study did not have any interference effects. These results indicate that the developed method can be applied satisfactorily for Cd determination in a variety of matrices.

### 3.5 Accuracy Check

Sewage Sludge-industrial origin (BCR no: 146R), NIST 1640a, and DOLT: 5 Dogfish Liver standard reference materials were analyzed to find the accuracy of the improved trap method for Cd. Under optimum conditions, three replicate measurements were carried out. The results for reference materials were in good agreement with the certified value, as given in Table 1.

**Table 1:** Determination of Cd in Standard Reference Materials Using the Proposed SQT-AT-FAAS Method

standard reference material	certified value	found value
BCR no: 146R (mg kg^–1^)	18.8 ± 0.5	19.9 ± 0.6
NIST 1640a (μg L^–1^)	3.992 ± 0.074	4.008 ± 0.052
DOLT: 5 (mg kg^–1^)	14.5 ± 0.6	14.8 ± 0.3

### 3.6 Analytical Figures of Merit

Calibration curves were plotted for all methods in this study. The linear portion of calibration for the SQT-FAAS method was 0.025–1.0 mg L^–1^. In this linear range, the correlation coefficient was found as 0.9998. LOD was calculated as three times the standard deviation of 11 measurements of the blank and divided by the slope of the working curve. For the SQT-FAAS method, LOD and LOQ (10s) were obtained to be 5.0 and 16.0 ng mL^–1^, respectively. It was determined that the trap method showed linearity between 0.25 and 2.0 ng mL^–1^. The best line equation and correlation coefficient were *y* = 0.3531*x* + 0.1454 and 0.9979, respectively. The volume of the analyte solution was also 29.6 mL. For the trap method, LOD and LOQ values were found to be 0.075 and 0.25 ng mL^–1^, respectively. The improvement factor for the LOD was found to be 64 and 1464 compared by SQT-FAAS and FAAS, respectively. The linear portion of calibration for the trap method was 0.25–2.0 μg L^–1^. In this linear range, the correlation coefficient was found as 0.9979. The analytical figures of merit which were found under the optimum experimental conditions are presented in Table 2 for FAAS, SQT-FAAS, and SQT-AT-FAAS methods.

**Table 2:** Comparison of Different Methods Used in This Study

	FAAS	SQT-FAAS	SQT-AT-FAAS
LOD, ng mL^–1^	110	5	0.075
LOQ, ng mL^–1^	366	16	0.25
linear range, μg L–^1^	250–5000	25–1000	0.25–2.0
calibration equation^a^	*y* = 0.0799[Cd]+0.0097	*y* = 1.6851[Cd]+0.013	*y* = 0.3531[Cd]+ 0.1454
sample volume, mL			29.6
trapping time, s			210

a *y* is the absorbance and [Cd] is the concentration of Cd in ng mL^–1^.

Comparison of the LOD values of the SQT-AT-FAAS method with those of ICP-OES, ICP–MS, ETAAS, and HGAAS methods is shown in Table 3. Table 3 reveals that LOD levels of the developed method are at the level of ICP–MS and ETAAS and lower than both levels of ICP–OES and HGAAS.

**Table 3:** Comparison of LOD Values of the Developed Methods with Other Methods

methods	LOD (ng mL^–1^)	references
ETAAS	0.20	(37)
ICP–MS	0.98	(38)
ICP–OES	0.2	(39)
HGAAS	0.7	(40)
SQT-AT-FAAS	0.075	this study

**Figure 5:**
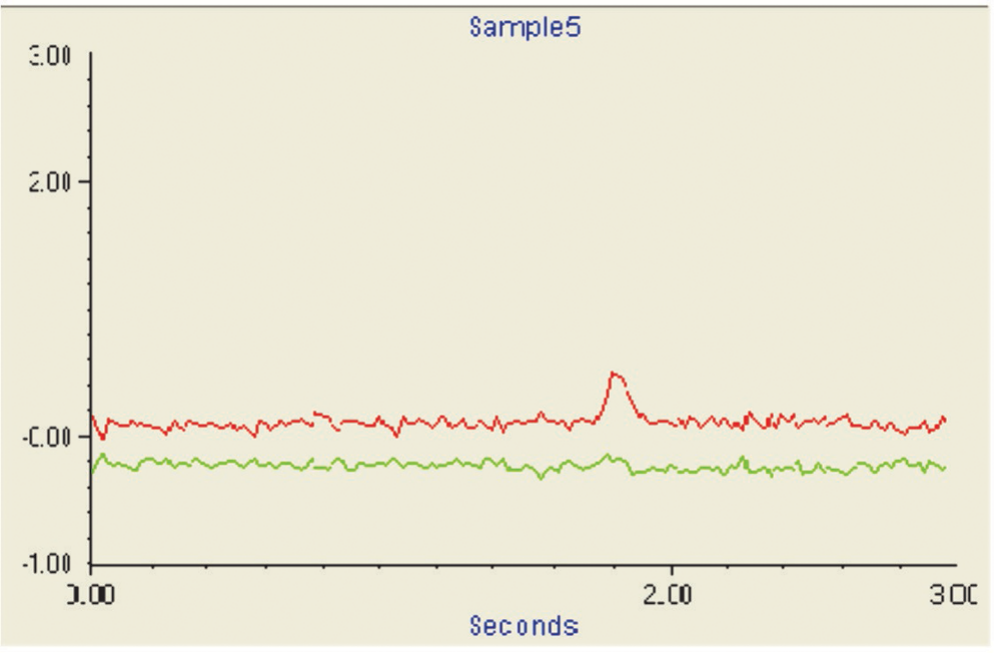
Analytical signal of Cd with a concentration of 5.0 ng mL^–1^ in the SQT-AT-FAAS method.

An analytical signal of Cd with a concentration of 5.0 ng mL in the SQT-AT-FAAS method is also shown in Figure 5.

### 3.7 Real Sample Analysis

The applicability of the proposed method on real samples was tested. For this purpose, the Cd concentration in some water and fish tissues was determined. High sensitivity was achieved by the SQT-AT-FAAS method under optimum conditions. The concentrations of Cd in real samples were determined successfully. Blank analysis of drinking water was first performed, and Cd was not detected under the optimum conditions. After that, drinking water samples were spiked at 0.5 and 1.5 ng mL^–1^ concentrations so as to cover the linear range. Precision, uncertainty, and accuracy of the proposed method are presented in Table 4. *t*-test was applied to compare the results obtained. *t*-test was calculated using Microsoft Excel using a 95% confidence level. The test statistic consists of comparing the *t*_critical_ with the *t*_calculated_. The *t*_critical_ value was 4.303. For each water sample, *t*_calculated_ values were calculated as 0.950, 0.470, 0.750, and 0.851. Since *t*_calculated_ values < *t*_critical_ value, the null hypothesis is accepted at the 95% confidence level, and it is concluded that there is no systematic error.

**Table 4:** Performance Characteristics of the Proposed Method (*n* = 3 for Standard Deviation)

sample	proposed method (ng mL^–1^)	added (ng mL^–1^)	found (ng mL^–1^)	error of measuremen*t* (ng mL^–1^)	error of measurement (%)	precision (SD)^a^ (ng mL^–1^)	precision (RSD %)^b^	measurement uncertainty^c^	accuracy (recovery) (%)
drinking water 1	<LOD	0.5	0.527	0.027	5	0.049	9.34	10.79	105
drinking water 2	<LOD	0.5	0.512	0.012	2	0.044	8.64	9.97	102
drinking water 3	<LOD	1.5	1.532	0.032	2	0.074	4.82	5.57	102
drinking water 4	<LOD	1.5	1.541	0.041	3	0.084	5.46	6.30	103

a Standard deviation.

b Relative standard deviation.

c *k* = 2, 95% confidence level.

The Cd concentrations of fish tissue samples are presented in Table 5. It was determined that Cd concentrations in liver tissue samples were higher than those of other tissue samples. Cd concentrations obtained for fish tissue samples were found to be below the maximum limit values determined by the European Union.

**Table 5:** Determination of Cd in Fish Samples by the SQT-AT-FAAS Method (*n* = 3 for Standard Deviation)

sample	muscle (μg kg^–1^)	liver (μg kg^–1^)	gill (μg kg^–1^)
red mullet 1	4.2 ± 0.4	9.3 ± 0.9	7.8 ± 0.5
red mullet 2	5.8 ± 0.6	10.5 ± 0.8	8.4 ± 0.8
red mullet 3	3.7 ± 0.4	7.7 ± 0.6	6.8 ± 0.6
common pandora 1	3.2 ± 0.3	7.4 ± 0.6	5.4 ± 0.5
common pandora 2	4.5 ± 0.3	8.2 ± 0.7	6.1 ± 0.6
common pandora 3	2.9 ± 0.4	6.8 ± 0.4	5.1 ± 0.4

## 4 Conclusions

In this study, the SQT-AT-FAAS method was developed as an atom trap for online preconcentration of Cd atoms. Although the same SQT, including all optimizations and calibrations, was used in experimental studies at least 300 times, there was no change in sensitivity at the end of the study. Analysis of standard reference materials was performed for accuracy check, and the Cd results were in good agreement with the certified values for the SQT-AT-FAAS method. The developed trap method was successfully applied to the determination of Cd in drinking water and fish tissue samples. In addition, transition metals, hydride-forming elements, and anions whose interference effects were examined did not show significant interference effects.

In summary, SQT-AT-FAAS was found to be a sensitive analytical method for Cd determination. The sensitivities are at the level of ICPMS, ETAAS, and HGAAS methods. The developed trap method for researchers who do not have relatively expensive instruments such as ICPMS, ICPOES, and AFS in their laboratories is a good alternative for Cd determination in environmental and food samples. In addition, the developed trap method in this study is simple, economical, and robust.
